# Efficacy of first-line systemic treatment in correlation with BRAF V600E and different KRAS mutations in metastatic colorectal cancer – a single institution retrospective analysis

**DOI:** 10.2478/v10019-011-0039-y

**Published:** 2011-11-16

**Authors:** Martina Rebersek, Marko Boc, Petra Cerkovnik, Jernej Benedik, Zvezdana Hlebanja, Neva Volk, Srdjan Novakovic, Janja Ocvirk

**Affiliations:** 1 Department of Medical Oncology; 2 Department of Molecular Diagnostics, Institute of Oncology Ljubljana, Ljubljana, Slovenia

**Keywords:** metastatic colorectal cancer, KRAS, BRAF, prognostic factors

## Abstract

**Background:**

KRAS mutation status in codons 12 and 13 is recognized as a predictive factor for resistance to anti-EGFR monoclonal antibodies. Despite having a wild type KRAS (wt-KRAS), not all patients with wt-KRAS respond to anti-EGFR antibody treatment. Additional mechanisms of resistance may activate mutations of the other main EGFR effectors pathway. Consequently, other molecular markers in colorectal cancer are needed to be evaluated to predict the response to therapy.

**Patients and methods:**

In this retrospective study, objective responses (OR), time to progression (TTP), overall survival (OS) were analyzed in 176 metastatic colorectal cancer (mCRC) patients treated with first-line chemotherapy in combination with monoclonal antibodies in respect of KRAS status in codons 12 and 13 and BRAF mutational status.

**Results:**

The KRAS mutations were found in 63 patients (35.8 %), the KRAS mutation in codon 12 in 53 patients (30.1%) and the KRAS mutation in codon 13 in 10 patients (5.7%). The BRAF V600E mutation was detected in 13 of 176 patients (7.4%). In the subgroup of mCRC patients having wt-KRAS and wild type BRAF (wt-BRAF), the objective response rates were higher (OR 54.0% ,CR 14.7%, PR 39.3%) than in the patients with wt-KRAS and mt-BRAF (OR 38.5%,CR 15.4%, PR 23.1%), the difference was not statistically significant (p= 0.378). Median OS in patients with wt-KRAS wt-BRAF, and in patients with wt-KRAS mt-BRAF, was 107.4 months and 45 months, respectively. The difference was statistically significant (p= 0.042). TTP in patients with wt-KRAS wt-BRAF, and in patients with wt-KRAS mt-BRAF, was 16 months and 12 months, respectively. The difference was not statistically significant (p= 0.558).

**Conclusions:**

Patients with BRAF V600E mutation have statistically significantly worse prognosis than the patients with wt-BRAF and progress earlier during treatment. The definitive role of the BRAF V600E mutation as a prognostic and predictive factor for the response to anti-EGFR monoclonal antibodies needs to be analyzed in large prospective clinical studies.

## Introduction

Colorectal cancer (CRC) is the fourth most common cancer and one of the leading causes of cancer death in the world. It is the most common cancer in Slovenia and, according to the Cancer Registry of Slovenia, 1279 new patients were diagnosed with CRC in 2007.^1^ The majority of patients need combined modality treatment and carful post-treatment surveillance is necessary to offer patient an optimal treatment approach.^2^,^3^ Metastatic disease is still incurable, with 5% five-year survival without treatment. With the introduction of new chemotherapy, using oxaliplatin and irinotecan in the current management of metastatic disease, in combination with biologicals, targeting epidermal growth factor- mediated growth regulatory pathway and the vascular endothelial growth factor-mediated angiogenesis pathway, we can prolong the progression-free survival (PFS) and overall survival (OS) of these patients.^4^–^8^ In selected patients with appropriate combination of therapy and surgery we can achieve approximately a 50% five-year survival.

The development of CRC is a multistep process which accumulates different gene mutations, chromosomal abnormalities and epigenetic changes.^9^,^10^ The mutations within KRAS proto-oncogen, predominately within codons 12 and 13, activate RAS/RAF signalling and are thought to occur early in carcinogenesis of CRC. The KRAS status is the first molecular marker to predict the response to anti-EGFR monoclonal antibodies cetuximab and panitumumab in metastatic CRC (mCRC) patients, and it needs to be determined before deciding in favor of treatment with anti-EGFR antibodies. As the KRAS mutations occur early in CRC formation, there is a high concordance between the KRAS mutations of primary tumour and metastases, which was confirmed in previous studies.^11^–^13^ In a recent retrospective study, de Roock with his colleagues raised the possibility that the patients with the KRAS mutation in codon 13 might have benefited from anti- EGFR antibodies treatment.^14^ The mutations in KRAS gene are found in approximately 30 to 40% of mCRC patients, reported in previous literature, but only 40 to 60% of these patients with wt-KRAS will respond to anti-EGFR antibodies treatment.^15^,^16^ Therefore, other molecular markers downstream of EGFR in the RAS/RAF/MAPK pathway and other effector pathways are found to be involved to predict the response to specific systemic therapy.

The BRAF gene encodes a serine/threonine protein kinase of the RAS/RAF/MEK/ERK kinase pathway and it is also involved in CRC carcinogenesis.^9^,^10^ The most common mutation of the BRAF gene is V600E which is found in approximately 5 to 9% of mCRC.^17^,^18^ The same was reported in our previous study carried on Slovenian patients with CRC where the BRAF V600E mutation was found in 5.1% of patients.^19^ Previous retrospective studies suggested that mt-BRAF was a marker of resistance to anti-EGFR therapy and that the patients with mt-BRAF had significantly shorter PFS and OS than the patients with wt-BRAF tumours.^20^ The mutations in the KRAS and BRAF genes have been reported to be mutually exclusive.^21^,^22^ In the retrospective analysis by Fariña- Sarasqueta *et al.*, it was also shown that the BRAF V600E mutation was an independent prognostic factor for the survival of patients with colon cancer in stages II and III, while the KRAS mutations did not have any effect on the overall survival of these patients. They concluded that the prognostic role of the KRAS mutations in an adjuvant setting has to be determined.^23^ In recent clinical studies, it was published that the BRAF V600E mutation in metastatic colorectal cancer is conferred to a poor prognosis regardless of treatment, but these patients may have some benefit from the treatment with cetuximab in combination with chemotherapy as the first-line therapy, but not when used in the patients in whom the disease has progressed after the first-line therapy.^17^

The aim of this retrospective study was to analyze objective responses, time to progression and overall survival of the patients with metastatic colorectal cancer treated with first-line systemic therapy in respect of KRAS and BRAF status.

## Patients and methods

### Patients

In the study, 176 patients with histologically confirmed metastatic colorectal cancer (mCRC), primarily metastatic or progressed during or after adjuvant therapy were retrospectively analyzed. They were treated according to the national and NCCN guidelines, including performance status of patients and comorbidity. They were treated with chemotherapy, including fluoropirimidins, capecitabine or 5-fluorouracil (5- FU), oxaliplatin or irinotecan in combination with biologicals, bevacizumab or cetuximab in respect of previously determined KRAS status. The treatment was continued according to the RECIST criteria, until the planned operation or until the progression of disease or toxicity occurred.

### Methods and assessment of response

All relevant data from medical files were collected and entered into the data base. Baseline data was analyzed with regard to age, sex, primary site (colon and rectum), number and location of metastases. Efficacy was evaluated according to the Response Evaluation Criteria in Solid Tumours (RECIST, version 1.1) by using computed tomografy (CT) scans, magnetic resonance scans, abdominal ultrasound, chest X-ray, bone scans, clinical examination and laboratory tests.^24^ The study was conducted in the conformance with the principles of the Declaration of Helsinki.

### Molecular analysis of KRAS and BRAF mutations

DNA for molecular analysis was extracted from formalin-fixed, paraffin-embedded tumour tissue of primary tumours or metastases with at least 70% of tumour cells. TheraScreen KRAS Mutation Kit^®^ (Roche Applied Science, Mannheim, D) was used to determine seven most common mutations in codons 12 and 13 of the KRAS gene. The V600E mutation in BRAF was detected by end-point genotyping using the TaqMan MGB probes (Applied Biosystems, Warrington, UK) as described previously.^19^ The mutation V600E in BRAF in positive tumour samples was confirmed by direct sequencing after amplification of the exon 15 of the BRAF gene.^19^

### Statistical analysis

The primary end-points of the analysis were overall response rate (ORR), based on RECIST criteria, overall survival (OS) and time to progression (TTP) according to the KRAS and BRAF status.

The χ^2^-test was used to compare ORR, OS and TTP between groups, with 95% confidence intervals (CI) calculated for the medians. OS and TTP were estimated by using Kaplan-Meier Estimates and compared using the log-rank test. TTP was measured in all patients from the beginning of the first-line systemic chemotherapy to the first evidence of progression. The duration of survival was calculated from the beginning of systemic treatment until the date of death. p value < 0.05 was considered statistically significant. Statistical data were obtained using the SPSS software package PASW statistics 18.0.

## Results

### Patients’ characteristics

In total, 176 patients with mCRC who received first-line therapy between May 2005 and October 2010 were included in the retrospective analysis. The cut-off date for the present analysis was April 2011. All patients were treated at the Institute of Oncology Ljubljana, all were Caucasian. The median age was 62 years (range 27–86 years) and the majority of the patients were males (61.4%). Most of the patients had metastatic colon cancer (71.4%). One hundred and four patients had primary metastatic disease (59.1%). The most common sites of metastases were liver and lung. The most common therapies the patients received were irinotecan, capecitabine with bevacizumab (29.5%) and oxaliplatin, capecitabine with cetuximab (22.1%). Twenty-four patients (13.6%) were treated only with chemotherapy, capecitabine in monotherapy, or with fluoropirimidines in combination with oxaliplatin or irinotecan. Patients’ baseline and disease characteristics are shown in Table 1.

KRAS mutations were found in 63 patients (35.8%), to be more precise, the KRAS mutation in codon 12 in 53 patients (84.0%) and the KRAS mutation in codon 13 in 10 patients (16.0%). The BRAF V600E mutation was detected in 13 of 176 patients (7.4%).

The mutations of the KRAS or BRAF gene were detected in total in 76 patients (43.4%) (Table1).

### Efficacy

**FIGURE 1:**
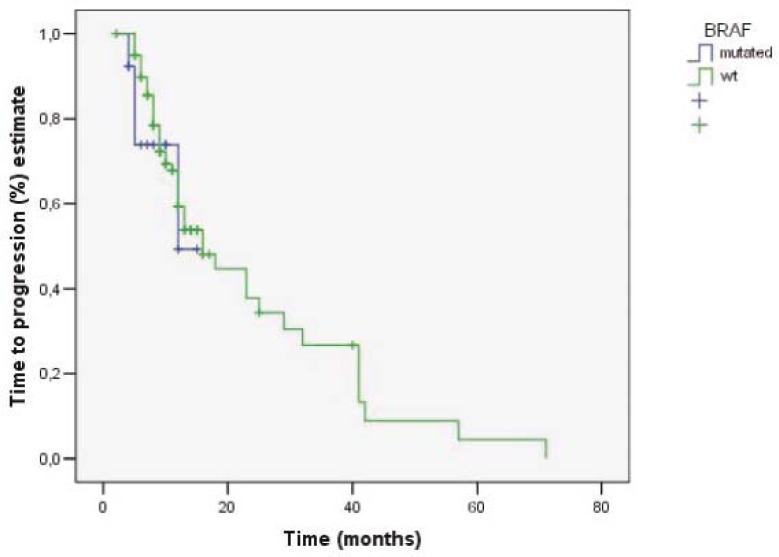
Time to progression in patients with wt-KRAS/wt-BRAF and wt-KRAS/mt-BRAF.

**FIGURE 2:**
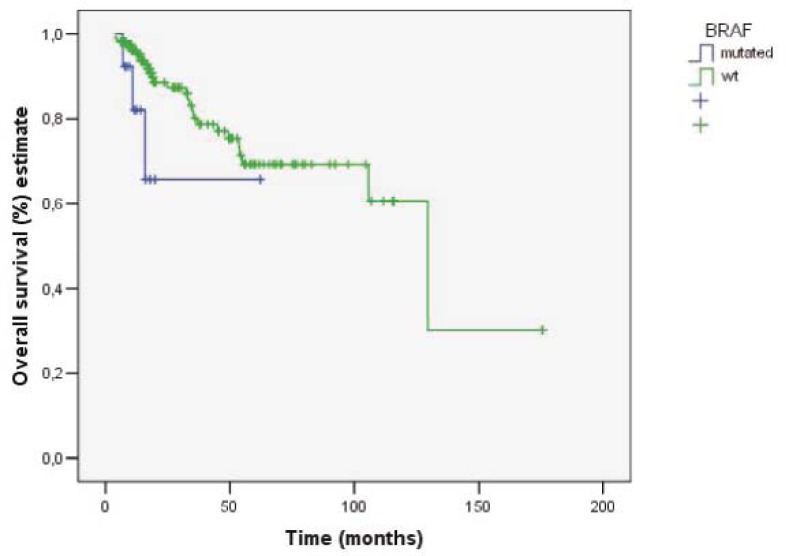
Overall survival in patients wt-KRAS/wt-BRAF and wt-KRAS/mt-BRAF.

The response rates according to RECIST criteria with regard to the KRAS and BRAF status are shown in Table 2. The overall response rates in patients with wt-KRAS and wt-BRAF and in patients with wt- KRAS and mt- BRAF were CR 14.7 % + PR 39.3 % + SD 35.5 % and CR 15.4 % + PR 23.1 % + SD 46.1 % respectively. The objective response rates in the group of patients with wt-KRAS and wt-BRAF tumours were 54.0% (CR 14.7%, PR 39.3%), while in the group of patients with wt-KRAS and mt-BRAF were 38.5% (CR 15.4%, PR 23.1%). The difference was not statistically significant (p= 0.378). The median OS in the group of patients with wt-KRAS and wt-BRAF tumours was 107.4 months (95% CI: 82- 132.9 months) and in the group of patients with wt-KRAS and mt-BRAF tumours 44.9 months (95% CI: 28.4- 61.5 months) (Figure 1). The difference in median OS between those two groups was statistically significant (p= 0.042). TTP in the group of patients with wt-KRAS and wt-BRAF tumours and in the group of patients with wt-KRAS and mt-BRAF tumours was 16 months (95% CI: 10.7- 21.2 months) and 12 months (95% CI: 4.0- 15.0 months), respectively (Figure 2). It was not statistically significant (p= 0.558).

In the KRAS mutation subgroups, the objective response rate of 53 patients with the mutation in codon 12 was 47% (CR 20.7%, PR 26.4%) and, in 10 patients with the mutation in codon 13, the objective response was 33% (CR 11.1%, PR 22.2%). The difference was not statistically significant (p= 0.08). TTP in the patients with the mutation in codon 12 and the patients with the mutation in codon 13 was 13.5 months (95% CI: 9- 18 months) and 9.3 months (95% CI: 5.1- 13.5 months), respectively. The difference was not statistically significant (p= 0.106).

Surgical resection of liver metastases was performed in 47/176 patients (26.7%); more specifically, in 31 patients with wt-KRAS tumours and in 16 patients with mt-KRAS tumours. R0 resection was achieved in 38/176 patients (21.6 %), of whom 37 patients had wt-BRAF and only one had mt-BRAF tumour.

## Discussion

In our study population, the KRAS mutations in codons 12 and 13 were found in 35.8% of patients, in most of them in codon 12; while the mutation V600E in BRAF gene was detected in 13 patients (7.4%). The results of testing are comparable with those previously reported, where the KRAS mutations were found in 30 to 40% and the BRAF V600E mutation in 5 to 9% of the patients.^11^–^13^,^17^–^19^

The presented data demonstrate that the patients with the BRAF V600E mutation have worse prognosis than the patients with wt-BRAF tumour and progress early during treatment. The patients with wt-BRAF tumours have higher response rates than the patients harbouring the BRAF V600E mutation, but the difference was not statistically significant. One third of the patients with wt-KRAS or mt-BRAF tumour still respond to the treatment¸ alluding that the BRAF status is not predictive for the response to anti-EGFR antibody therapy. This was also reported in previously published analyses and, in recently published retrospective meta-analysis of the CRYSTAL and OPUS studies, it was also concluded, that the patients with BRAF mutation might have also benefited from the treatment with anti-EGFR antibodies.^17^,^18^,^21^–^23^,^25^ At this point it should be highlighted that not all patients in our retrospective analysis with wt-KRAS received cetuximab-based first-line systemic therapy; the therapy was selected in accordance to the patients’ baseline characteristics, the purpose of treatment or planned operation for metastases.

The difference in TTP between the patients with wt-KRAS and wt-BRAF tumours and the patients with wt-KRAS and mt-BRAF tumours was 4 months. The difference was not statistically significant, probably due to our small group of patients and, consequently, small proportion of the patients with BRAF mutation. The comparison of median OS of those two groups showed a statistically significant difference which was also accompanied with a better prognosis of patients with wt-KRAS and wt-BRAF tumour. These results are comparable with those reported earlier.^17^,^20^,^21^ The results of retrospective pooled analysis from randomized CRYSTAL and OPUS trials showed that cetuximab as the first-line chemotherapy based on irinotecan or oxaliplatin significantly improved OS, ORR and PFS the in patients with wt-KRAS tumours. According to the results of the same meta-analysis, the patients with BRAF mutations also appeared to have benefited from cetuximab as the first-line systemic treatment.^25^,^26^

In our retrospective study, the KRAS mutations were most frequently detected in codon 12. This is in accordance with the results of our previous study.^19^ Comparing the patients having KRAS mutations in codon 12 with the ones having the mutation in codon 13 after the treatment with chemotherapy and bevacizumab, the response rates were higher in the patients with the mutations in codon 12. Nevertheless, the differences in response rates, OS and TTP between these two groups were not statistically significant; we assume that the groups of patients were too small. In the contrast, in their retrospective study, De Roock *et al.* showed that the patients with the mutation in codon 13 KRAS who were treated with cetuximab had better overall and progression-free survival than the patients with other KRAS mutations and might have benefited from the treatment with cetuximab.^14^ In an abstract recently published in the 2011 ASCO Annual Meeting Proceedings, Tejpar *et al.* retrospectively analyzed the influence of KRAS G13D mutations on the efficacy of treatment with cetuximab as the first-line systemic therapy and compared it with the pooled results of randomized studies CRYSTAL and OPUS. The patients with the KRAS mutation in codon 13 had a much lower treatment effect compared to the patients with wt-KRAS tumours and might have nevertheless benefited from treatment with cetuximab.^27^

Although not studied in our retrospective analysis, other KRAS mutations were also reported to predict the response to anti- EGFR monoclonal antibodies. The results of a small study of 74 patients, conducted by Loupakis with his colleagues, suggested that rare KRAS mutations in codon 61 and in codon 146 might also be responsible for in the treatment resistance to anti-EGFR monoclonal antibodies.^28^,^29^ In contrast, in their large retrospective analysis, De Roock *et al.* concluded that the codon 146 mutations did not affect the response to cetuximab and that the patients with codon 61 mutant tumours had lower response rate.^20^ According to the analysis of other mutations, they proposed testing of KRAS status, if not mutated, then of BRAF and NRAS status, and PIK3CA exon 20 mutation in order to improve the objective response up to 40% in selected patients.

In our retrospective study, 26.7% of patients, all with KRAS wild-type tumours, who had previously unresectable liver-only metastases, underwent surgical resection after systemic therapy, with R0 resection achieved in 38 patients (21.6%); one of those was patient with the BRAF V600E mutation. Although it is difficult to make any comparison, because our patients were not selected according to specific systemic therapy, these results are comparable with those reported in previous studies claiming that 19 to 23% patients treated with bevacizumab- and irinotecan-based chemotherapy and with previously unresectable liver-only metastases underwent resection.^30^–^32^ In a recently published clinical study BOXER, where the patients with unresectable liver-only metastases were treated with oxaliplatin, capecitabine and bevacizumab, R0 resection was achieved in 40% of patients.^33^ The proportion of patients with resected liver metastases in our retrospective study was higher than that reported in earlier studies including the patients with previously unresectable liver-only metastases and treated with cetuximab in combination with irinotecan- or oxaliplatin-based chemotherapy; resection was achieved in 4 to 10%.^34^,^35^ In the randomized phase II CELIM study, in which the patients with liver-only metastases were treated with irinotecan- or oxaliplatin-based chemotherapy with cetuximab as the first-line systemic therapy, the proportion of R0 resection was higher; it was achieved in 34% of patients.^36^ In another phase II POCHER trial, the proportion of R0 resection was even higher; it was achieved in 60% of patients who were treated with chronomodulated chemotherapy with irinotecan, oxaliplatin, 5- fluorouracil and leucovorin.^37^

In conclusion, the results of our retrospective study showed that the patients with BRAF V600E mutation had worse prognosis than those with wt-BRAF, with lower response rates and progressed early during systemic treatment, consequently, with less possibilities to achieve resectability of metastatic disease. The definitive role of the BRAF V600E mutation as a prognostic and predictive factor to response to the anti-EGFR monoclonal antibodies needs to be analyzed in large prospective clinical studies. Different KRAS mutations in codon 12 and 13 and other molecular markers, predictive or prognostic, downstream of EGFR in the RAS/RAF/MAPK pathway, and other effector pathways, are needed to be defined to select the patients, who will benefit from specific systemic therapy in a way of individualized treatment.

## Figures and Tables

**TABLE 1:** Baseline and disease characteristic of patients

**Caracteristics**	**Patients, n= 176 (%)**
Gender	
Male	108 (61.4)
Female	68 (38.6)
Age(years)	
Median	62
Range	(27– 86)
WHO PS*	
0	126 (71.6)
1	50 (28.4)
Primary tumour localization,	
Colon	125 (71)
Rectum	51 (28)
Metastatic site	
Liver	68 (38.6)
Lung	11 (6.3)
Liver and lung	12 (6.8)
Other	85 (49.3)
KRAS status	
KRASw	113 (64.2)
KRASm 12	53 (84.0)
KRASm 13	10 (16.0)
BRAF status	
BRAFw	163 (92.6)
BRAFm	13 (7.4)

* WHO PS- World Health Organization performance status

**TABLE 2:** Response rates in KRAS wild type patients according to BRAF status in first-line therapy

	**wKRAS**	**wKRAS/wBRAF**	**wKRAS/mBRAF**
Overall response rate (CR+ PR), n (%)	93 (52.8)	88 (54.0)	5 (38.5)
Disease control rate (CR+PR+SD), n (%)	157 (89.2)	146 (89.5)	11 (84.6)
CR	26 (14.8)	24 (14.7)	2 (15.4)
PR	67 (38.0)	64 (39.3)	3 (23.1)
SD	64 (36.4)	58 (35.5)	6 (46.1)
PD	19 (10.8)	17 (10.5)	2 (15.4)
Median OS, months estimate	129.4 (95% CI: 52.4- 206.4)	107.4 (95% CI: 82- 132.9)*	44.9 (95% CI: 28.4- 61.5) *
Median TTP, months estimate	15.9 (95% CI: 10.8- 21.0)	16.0 (95% CI: 10.7-21.2)**	12.0 (95% CI: 4.0-15.0) **

* p= 0.042

** p= 0.558
